# A deep semantic segmentation correction network for multi-model tiny lesion areas detection

**DOI:** 10.1186/s12911-021-01430-z

**Published:** 2021-07-30

**Authors:** Yue Liu, Xiang Li, Tianyang Li, Bin Li, Zhensong Wang, Jie Gan, Benzheng Wei

**Affiliations:** 1 Center for Medical Artificial Intelligence, Shandong University of Traditional Chinese Medicine, Qingdao, 266112 China; 2 Qingdao Academy of Chinese Medical Sciences, Shandong University of Traditional Chinese Medicine, Qingdao, 266112 China; 3 College of Intelligence and Information Engineering, Shandong University of Traditional Chinese Medicine, Jinan, 250355 China; 4 Radiology Department, Second Affiliated Hospital of Shandong University of Traditional Chinese Medicine, Jinan, 250001 China

**Keywords:** White matter hyperintensities, Focal cerebral ischemia, Lacunar infarct, Magnetic resonance imaging, Multi-modality

## Abstract

**Background:**

Semantic segmentation of white matter hyperintensities related to focal cerebral ischemia (FCI) and lacunar infarction (LACI) is of significant importance for the automatic screening of tiny cerebral lesions and early prevention of LACI. However, existing studies on brain magnetic resonance imaging lesion segmentation focus on large lesions with obvious features, such as glioma and acute cerebral infarction. Owing to the multi-model tiny lesion areas of FCI and LACI, reliable and precise segmentation and/or detection of these lesion areas is still a significant challenge task.

**Methods:**

We propose a novel segmentation correction algorithm for estimating the lesion areas via segmentation and correction processes, in which we design two sub-models simultaneously: a segmentation network and a correction network. The segmentation network was first used to extract and segment diseased areas on T2 fluid-attenuated inversion recovery (FLAIR) images. Consequently, the correction network was used to classify these areas at the corresponding locations on T1 FLAIR images to distinguish between FCI and LACI. Finally, the results of the correction network were used to correct the segmentation results and achieve segmentation and recognition of the lesion areas.

**Results:**

In our experiment on magnetic resonance images of 113 clinical patients, our method achieved a precision of 91.76% for detection and 92.89% for classification, indicating a powerful method to distinguish between small lesions, such as FCI and LACI.

**Conclusions:**

Overall, we developed a complete method for segmentation and detection of WMHs related to FCI and LACI. The experimental results show that it has potential clinical application potential. In the future, we will collect more clinical data and test more types of tiny lesions at the same time.

## Background

White matter hyperintensities (WMHs) are features of very small vessel disease of the brain [1, 2], which they present as hyperintense regions on fluid-attenuated inversion recovery (FLAIR) images. Accurately identifying and classifying WMHs can help radiologists diagnose diseases and determine the course of such diseases [3, 4].

Among them, detection and recognition for WMHs related to focal cerebral ischemia (FCI) and lacunar infarction (LACI) signals are of great significance for the clinical diagnosis and prevention of lacunar cerebral infarction. FCI presents high value signals on T2 FLAIR images and no obvious signal on T1 FLAIR images. These abnormal signals are mainly caused by demyelination [5, 6]. In patients who experienced an ischemic event, treating the underlying cause in time is critical for the prevention of further episodes. If its duration is long enough, FCI will lead to irreversible brain tissue necrosis or infarction in the ischemic areas [7]. In other words, FCI can generally be cured, while infarction cannot. LACI is a common type of infarction. Patients considered to have had LACI usually present high value signals similar to those observed in FCI on T2 FLAIR images and low value signals on T1 FLAIR images. These abnormal signals are caused by demyelination and structural changes. Thus, patients with LACI who undergo diagnostic imaging should be educated on common stroke symptoms and how to manage the onset of stroke [8]. In addition, continuous follow-up with a physician is necessary for these patients so that the physician can monitor drug dosage and risk factors [9]. Therefore, it is important to recognize between these two abnormal signals.

Because the signals of FCI and LACI are indistinguishable on T2 FLAIR images, diagnosis with both T1 FLAIR and T2 FLAIR images is usually required [10]. Clinically, the conventional method of finding and distinguishing focal abnormal signals from a patient relies on careful examination by multiple radiologists. The manual method is time-consuming and can easily cause missed diagnosis and misdiagnosis, especially after doctors review a large number of radiological images at once. A practical tool that can assist radiologists in finding and distinguishing focal abnormal signals is urgently needed.

**Fig. 1:**
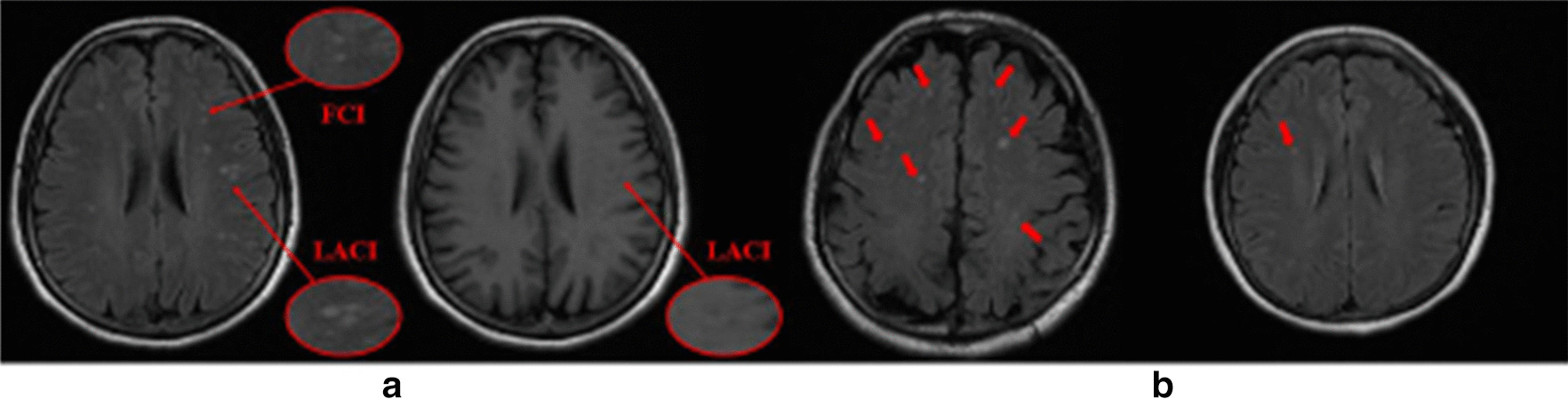
**a** Comparison of FCI and LACI signals on T2 FLAIR and T1 FLAIR images.This patient had both of these lesions on the same slice. It is observed that the signals of FCI and LACI can only be distinguished on T1 FLAIR images. **b** Two slices with strong differences in the number and brightness of abnormal signals. These differences make it difficult for the segmentation model to accurately segment both types of slices at the same time

However, it is a challenge to accurately detect and distinguish focal abnormal signals of the brain. Although many researchers have worked on lesion segmentation, the solution of cerebral focal abnormal signal segmentation lacks relevant experience. First, focal abnormal signals are always tiny objects and are difficult to detect accurately. Existing solutions of medical image analysis often underperform with tiny objects. Second, lesions cannot be accurately diagnosed using single-modal magnetic resonance imaging (MRI) data; however, how to apply multi-modal MRI data effectively is a challenge. Most of the existing multi-modal methods use feature fusion, which is not suitable for focal abnormal signal segmentation. Figure 1a shows the T1 and T2 FLAIR images of a patient with both FCI and LACI. In clinical diagnosis, radiologists first observed the T2 FLAIR images for focal abnormal signals and then compared these images at the same location on T1 FLAIR images. To our knowledge, existing methods have never been explored in a similar process. Finally, the size and number of lesions vary greatly for different subjects. Figure 1b shows a comparison of two patients with focal abnormal signals on T2 FLAIR images. It is difficult to use the same model to intelligently determine the number of lesions in a different subject.

To overcome the aforementioned challenges, we herein propose a new framework to estimate the lesion areas via segmentation and correction processes, in which we simultaneously train two models: a segmentation network and a correction network. The segmentation network was first used to extract and segment potentially diseased areas on T2 FLAIR images. The correction network was then used to classify these areas at the corresponding locations on T1 FLAIR images to assess the probability that a patient has had LACI. Through the combination of these two networks, we achieved semantic segmentation of two different lesions.

## Related work

### Segmentation of white matter hyperintensities

Most related work focuses on automate segmentation of WMHs. The abnormal signals of FCI and LACI also belong to WMHs but are difficult to use for classifying and grading lesions. Moreover, all the existing work focuses on abnormal signals with large areas, and there is a lack of exploration on the segmentation of abnormal signals with very small areas. These methods can be divided into unsupervised, semi-supervised, and supervised methods.

#### Unsupervised segmentation

The advantage of unsupervised segmentation methods is that manual labeling is not required. Most of these methods use intensity-based clustering methods, such as fuzzy C-means methods [11], EM-based algorithms [12], and Gaussian mixture models [13]. Some studies have designed probabilistic generative models for stroke lesion segmentation, such as those proposed in reference numbers [14, 15]. Additionally, several studies have focused on the fact that WMHs are best observed on FLAIR magnetic resonance images and identified differently on T1-weighted magnetic resonance images [16, 17]. These studies generated synthetic images and then compared them with real FLAIR images to detect any abnormalities. An important disadvantage of these methods is that they are not designed to find and distinguish between FCI and LACI. Therefore, these methods cannot accurately segment the diseased area nor can they distinguish whether the infarct has occurred in the segmented area.

#### Semi-supervised segmentation

Existing semi-supervised segmentation methods mainly depend on regional growth techniques. Kawata et al. [18] proposed a region-growing method, which was adaptive selection on WMH regions based on a support vector machine. Qin et al. [19] proposed an algorithm to optimize the kernel-based max-margin objective function. Although these methods are well motivated and have yielded some progress, transferring useful knowledge from unlabeled data remains a challenge. Therefore, semi-supervised WMH segmentation methods cannot completely replace supervised methods.

#### Supervised segmentation

Recently, a variety of convolutional neural networks (CNNs) have been widely utilized in the medical field and have often been reported to be the state of the art [20–22]. Guerrero et al. [23] used a CNN that is able to segment hyperintensities and differentiate between WMHs and stroke lesions. Brosch et al. [24] proposed a deep convolutional encoder network for the prediction of MS lesions. Kamnitsas et al. [25] proposed a dual-pathway 3D CNN for brain lesion segmentation. Ghafoorian et al. [26] proposed several deep CNN architectures to consider multi-scale patches or take explicit location features. However, none of these methods can achieve semantic segmentation of WMHs related to FCI and LACI.

### Tiny objects

Processing tiny objects is notoriously challenging. The most common methods of distinguishing tiny objects are increasing the input image resolution [27] and fusing high-resolution features from low-resolution images [28, 29]. However, these methods greatly increase the computational overhead and do not address the class imbalance between tiny objects and backgrounds. Li et al. [30] proposed a perceptual generative adversarial network (PGAN). The PGAN lifts representations of tiny objects to “super-resolved” ones, achieving characteristics similar to those of large objects. Improving tiny object proposals was proposed on the basis of different resolution layers in a region proposal network [31]. Ren et al. [32] leveraged the context information and thereby further improving the performance of tiny object detection.

### Our contributions

The main contributions of this research are as follows:

- For the first time, we achieved accurate segmentation of and discrimination between FCI and LACI signals.
- We proposed a novel auxiliary network for discriminating between FCI and LACI signals in the T1 FLAIR modality.
- We proposed a series of oversampling and augmentation strategies to achieve tiny lesion segmentation.

## Methods

**Fig. 2:**
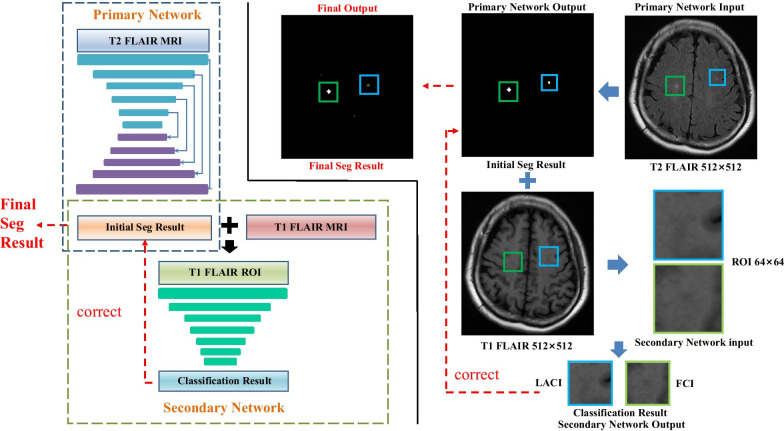
Segmentation correction network

Our proposed framework consisted of a primary network and a secondary network, as shown in Fig. 2. The primary network was used to focus on the segmentation of tiny brain lesions. Thereafter, the secondary network was used to receive the ROI that was extracted either from FCI masks or LACI masks and to output a scalar of 0 or 1 for each ROI to identify the type of lesion. Finally, the results of the secondary network were used to correct the segmentation results. Besides, to enable the model to process magnetic resonance images of different courses at the same time, we used a series of oversampling and augmentation strategies, each designed for the characteristics of tiny lesion segmentation in the brain.

### Primary network

**Fig. 3:**
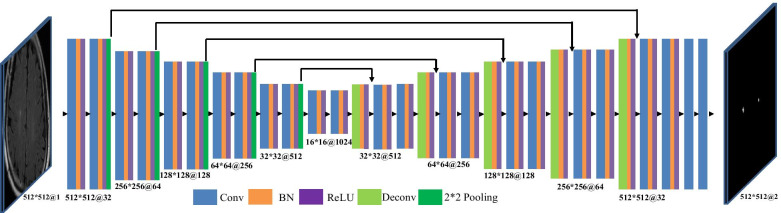
Primary network: segmentation network

To segment the lesions accurately and reliably, we deployed a primary network based on T2 FLAIR images. The proposed primary network had a symmetric model structure, as shown in Fig. 3. U-Net [33] forms the foundation of this architecture. It consisted of an encoding path and a decoding path. The encoding path comprised 10 convolutions with a kernel size of 3 $\times$ 3 for generating a set of feature maps. These maps were applied by batch normalization (BN) and a rectified linear unit (ReLU). After each two continuous stack of convolution + BN + ReLU, a 2 $\times$ 2 max-pooling layer was applied for down-sampling. Each decoding path had an up-sampling process of the feature map with a 2 $\times$ 2 deconvolution that halved the number of feature channels, each followed by a BN layer and a ReLU layer; each feature map was connected to the coding path after up-sampling. Thereafter, further feature extraction and selection were conducted for the concat feature map-based continuous convolution + BN + ReLU operations. At the final process, two continuous 1 $\times$ 1 convolutions were applied for mapping each component eigenvector to the required number of classes. When the size of the feature map was constant, two continuous 1 $\times$ 1 convolutional layers were used to increase the depth of the main network and significantly increase the nonlinear characteristics.

### Secondary network

**Fig. 4:**
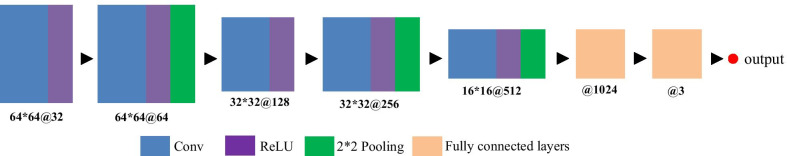
Secondary network: semantic correction network

Although the primary network segmented the lesions, semantic segmentation of WMHs related to FCI and LACI had not yet been implemented. We deployed the secondary network to implement this challenge based on T1 FLAIR images. The proposed secondary network comprised five convolutional layers with 3 $\times$ 3 kernels, five ReLUs, three average pooling layers, and two fully connected layers with dropout, as shown in Fig. 4. After each two continuous convolution + BN + ReLU layers, a 2 $\times$ 2 max-pooling layer for down-sampling was applied. At the final process, two continuous 1 $\times$ 1 convolutional layers were used to discriminate the class of the entire import image. With regard to training, the secondary network first received either the predicted FCI masks or the LACI masks from the primary network. Thereafter, the secondary network outputted a single scalar to determine whether the predicted masks had the characteristics of FCI or LACI. When the secondary network successfully discriminated the type of input image, this result was returned to correct the segmentation results, and the semantic segmentation result was then generated.

### Global optimization

The updates of our network were based on the cross-entropy loss function. For optimization, we used the RMSProp algorithm [34] as follows:

1 $$\begin{aligned} E[g^2]_{t}= & {} {\alpha }E[g^2]_{t-1} + (1 - {\alpha })g^{2}_{t}, \end{aligned}$$

2 $$\begin{aligned} W_{t+1}= & {} W_{t} - \frac{\eta }{\sqrt{E[g^2]_{t} + \epsilon }} {\odot } g_{t}, \end{aligned}$$

where $g_t$ denotes the gradient of the cost function; $E[g^2]_t$ denotes the gradient’s mean value of *t* times square; $\alpha$ is the moving average parameter set to 0.9; $\eta$ is the base learning rate set to 0.01; and $\epsilon$ is a parameter added to prevent division by zero.

## Data and experiments

### Original data and ground truth

The proposed framework was evaluated on a dataset of 113 clinical patients (61 men and 52 women). The average age of the patients was 52 ± 26 years. All MRI data were acquired using the GE Signa Horizon HDxt 1.5T clinical scanner (General Electric, Milwaukee, WI, USA), with a self-shielding gradient set at a maximum of 33 mT/m and an 8-channel phased-array head coil. The MRI examination comprised T1 FLAIR (TE = 23 ms, TR = 1750 ms) and T2 FLAIR (TE = 155 ms, TR = 8500 ms). The field-of-view of all sequences was 512 $\times$ 512 mm, and the slice thickness was 6 mm. The dataset contained 30 patients with LACI (70 images), 68 patients with FCI (191 images), and 37 healthy patients (152 images). According to the imaging diagnostic criteria, the training set was labeled with the boost of two experienced radiologists. The basic facts of the classification were extracted from clinical reports and reviewed by two doctors.

### Oversampling and augmentation strategies

The lesion region of WMHs related to FCI and LACI is usually very small and hard to recognize. In the experiment, we found that if we simply select images randomly for training, the model would classify all pixel points into negative pixels regardless of how the network sets the objective weight. According to Van Nguyen et al. [17], we speculate that this result is attributed to two factors: (1) Fewer images contain lesions, and (2) the lesions are not apparent in the image containing them. To solve this problem, we designed oversampling and augmentation strategies to encourage the model to focus on lesion regions.

#### Oversampling

We addressed the issue of relatively fewer images containing lesions by oversampling those images during training [35]. Based on the characteristics of tiny objects, we identified the slices with a large number of tiny lesions for pre-training. After the model converges, we input all the data used for training to the network.

#### Dataset augmentation

To focus on lesion segmentation, we introduced a dataset augmentation strategy. After careful confirmation by two radiologists on different MRI modalities, we finally selected 48 slices that had more than 5 WMHs related to FCI and LACI from 113 clinical patients. Each slice was rotated 5$^\circ$ to 10$^\circ$ clockwise or counterclockwise. All images and labels were shuffled and checked one by one to ensure that they had no errors after data augmentation.

#### Images used by secondary network

The images used by the secondary network were derived from the centroids of each connected component of the segmentation mask. Each image was an ROI from a centroid with a size of 32 $\times$ 32. To facilitate the calculation, we up-sampled the image to a size of 64 $\times$ 64 before being used as the input to the secondary network.

Owing to the very small size of these extracted images, the difference between the minimum and maximum values of the pixel intensity was within 100. To make the features of these images easier to extract, we leveraged the gamma transformations of the image intensity. To moderately stretch the pixels with high-intensity levels in the image and compress the pixels with low-intensity levels, we set the $\gamma$ value to 1.5.

### Experiments

We used the standard five-fold cross-validation method for the performance evaluation of our proposed method [36]. We divided the data into five parts, each time selecting four groups for training and one group for testing. When all the experiments were completed, we calculated the average value as the model metrics. Finally, all slices were used for training and testing, and each slice was used for testing only once. We implemented the proposed method using Python 3.7 based on the TensorFlow 1.13 library on a workstation equipped with GPUs of NVIDIA TESLA V100.

### Performance evaluation

We evaluated our proposed method in three dimensions: segmentation, detection, and classification. Among them, the evaluation index of segmentation was the dice coefficient, and that of detection and classification was precision. Precision was utilized to evaluate the accuracy of the results. In general, precision is defined as the ratio of true positives to all positives. A correct detection is only counted as a true positive detection if the predicted mask or bounding box has an intersection over union (IoU) higher than 0.6. In this study, our primary interest was precision in distinguishing each lesion region.

## Results

We proposed a novel method for segmentation of WMHs related to FCI and LACI. In our experiment on 113 sets of clinical patients’ MRI scans, our method achieved a precision of 91.76% for detection and 92.89% for classification. The results demonstrated the validity of the proposed method for semantic segmentation of WMHs related to FCI and LACI.

To investigate the effectiveness of our proposed method, we compared the experimental results with those from other networks. We analyzed the effects of the secondary network, oversampling, and augmentation on the detection results. Table 1 provides the experimental configurations and results. The results showed that our proposed method could distinguish the WMHs related to FCI and LACI effectively. Notably, there is no classification metric for the primary network in Table 1 because it only completes semantic segmentation according to the lesion area.

**Table 1:** Effectiveness of our proposed method on the dice coefficient, precision, for methods including the primary network, two-stage network, and augmentation(Aug) + oversample strategies

Method	Task	Segmentation	Detection	Classification
Primary network	a	10.23	9.25	–
	b	32.92	16.12	–
Primary network + Aug	a	15.83	12.96	–
	b	29.56	15.80	–
Primary network + Oversample	a	34.66	24.07	–
	b	54.39	58.06	–
Primary network + Aug + Oversample	a	32.48	52.18	-
	b	67.50	80.64	–
Two-stage network	b	26.53	28.23	66.97
Two-stage network + Aug	b	30.17	58.40	75.21
Two-stage network + Oversample	b	67.82	76.47	87.45
Proposed method	b	**74.21**	**91.76**	**92.89**

Task a: segmentation by category information (e.g., background, FCI and LACI); Task b: segmentation by lesion area (e.g., background and lesion area)

## Discussion

The experimental results of this study are presented in Table 1. The experiment is similar to an ablation study previously conducted to demonstrate the effect of each novel module. In the experiment, each index of the primary network had two results. This is because in the early stage of this research, we first thought that the processes of segmentation and classification were completed simultaneously; however, the results obtained were poor (reference to task a in Table 1 for more details). Although the introduction of data augmentation and oversampling strategy improved the results in the later stage, it still could not meet the requirements of high-precision segmentation and recognition. Therefore, we introduced a two-stage learning strategy, in which the primary network was only focused on segmenting the lesion areas, and the task of identifying the lesions was completed by the secondary network (reference to task b in Table 1 for more details). This two-stage network originated from clinical practice and completely simulated the diagnostic process used by radiologists. The final results also proved the effectiveness of our two-stage network. After deployment of the secondary network, the model significantly improved the classification effect for FCI and LACI. In addition, the experiments showed that oversampling is necessary for tiny lesions, which may be attributed to the small proportion of the lesions. Data augmentation technology effectively improves the ability of the model to detect lesions. This shows that in future research, collecting more data may be the key point for improving the accuracy of the model.

## Conclusions

In this study, we developed a complete method for segmentation of WMHs related to FCI and LACI. This is the first method to distinguish between small lesions, such as FCI and LACI. The experiments with 113 sets of clinical data showed that our method is accurate and reliable. This method first leverages the primary network to achieve segmentation of the lesions. Thereafter, the secondary network is deployed to classify the lesion type. Although existing studies have achieved semantic segmentation of multiple lesions, some of them (e.g., tiny lesions) have not been fully considered. In the future, we will collect more clinical data and test more types of tiny lesions at the same time.

## Data Availability

The datasets used and/or analyzed during the current study are available from the correspondence author on reasonable request.

## Abbreviations

FCI: Focal cerebral ischemia
LACI: Lacunar infarction
WMHs: White matter hyperintensities
FLAIR: Fluid-attenuated inversion recovery
IoU: Intersection over union
MRI: Magnetic resonance imaging
CNN: Convolutional neural networks
BN: Batch normalization
ReLU: Recitified linear unit
TE: Time of echo
TR: Time of repeatation
ROI: Region of interest

## References

[1] Wardlaw JM, Pantoni L. Sporadic small vessel disease: pathogenic. Cereb Small Vessel Dis. 2014;52.

[2] Lee Y, Ko J, Choi YE, Oh JS, Kim JS, Sunwoo MK, Yoon JH, Kang SY, Hong JY (2020). Areas of white matter hyperintensities and motor symptoms of Parkinson disease. Neurology.

[3] Birenbaum A, Greenspan H. Longitudinal multiple sclerosis lesion segmentation using multi-view convolutional neural networks. In: Deep learning and data labeling for medical applications, pp. 58–67. Springer, Cham; 2016.

[4] Moscoso A, Rey-Bretal D, Silva-Rodríguez J, Aldrey JM, Cortés J, Pías-Peleteiro J, Ruibal Á, Aguiar P, Initiative ADN (2020). White matter hyperintensities are associated with subthreshold amyloid accumulation. NeuroImage.

[5] Bokde A, Teipel S, Zebuhr Y, Leinsinger G, Gootjes L, Schwarz R, Buerger K, Scheltens P, Moeller H-J, Hampel H (2002). A new rapid landmark-based regional MRI segmentation method of the brain. J Neurol Sci.

[6] De Leeuw F, de Groot JC, Achten E, Oudkerk M, Ramos L, Heijboer R, Hofman A, Jolles J, Van Gijn J, Breteler M (2001). Prevalence of cerebral white matter lesions in elderly people: a population based magnetic resonance imaging study. The Rotterdam scan study. J Neurol Neurosurg Psychiatry.

[7] Front matter. In: Caplan LR, Biller J, Leary MC, Lo EH, Thomas AJ, Yenari M, Zhang JH (eds.) Primer on cerebrovascular diseases, 2nd edn. Academic Press, San Diego; 2017. 10.1016/B978-0-12-803058-5.01001-8

[8] Cardoso MJ, Sudre CH, Modat M, Ourselin S. Template-based multimodal joint generative model of brain data. In: International conference on information processing in medical imaging. Springer; 2015. p. 17–29. 10.1007/978-3-319-19992-4_2 26221664

[9] Arboix A, Martí-Vilalta JL (2009). Lacunar stroke. Expert Rev Neurother.

[10] Sapkota A, Lee C-H, Park SJ, Choi JW (2020). Lysophosphatidic acid receptor 5 plays a pathogenic role in brain damage after focal cerebral ischemia by modulating neuroinflammatory responses. Cells.

[11] Gibson E, Gao F, Black SE, Lobaugh NJ (2010). Automatic segmentation of white matter hyperintensities in the elderly using flair images at 3t. J Magn Reson Imaging.

[12] Dugas-Phocion G, Ballester MAG, Malandain G, Lebrun C, Ayache N. Improved em-based tissue segmentation and partial volume effect quantification in multi-sequence brain MRI. In: International conference on medical image computing and computer-assisted intervention. Springer; 2004. p. 26–33.

[13] Freifeld O, Greenspan H, Goldberger J (2009). Multiple sclerosis lesion detection using constrained GMM and curve evolution. Int J Biomed Imaging.

[14] Forbes F, Doyle S, Garcia-Lorenzo D, Barillot C, Dojat M. Adaptive weighted fusion of multiple MR sequences for brain lesion segmentation. In: 2010 IEEE international symposium on biomedical imaging: from nano to macro. IEEE; 2010. p. 69–72.

[15] Derntl A, Plant C, Gruber P, Wegener S, Bauer JS, Menze BH. Stroke lesion segmentation using a probabilistic atlas of cerebral vascular territories. In: BrainLes 2015. Springer; 2015. p. 21–32.

[16] Bowles C, Qin C, Ledig C, Guerrero R, Gunn R, Hammers A, Sakka E, Dickie DA, Herndez MV, Royle N, et al. Pseudo-healthy image synthesis for white matter lesion segmentation. In: International workshop on simulation and synthesis in medical imaging. Springer; 2016. p. 87–96.

[17] Van Nguyen H, Zhou K, Vemulapalli R. Cross-domain synthesis of medical images using efficient location-sensitive deep network. In: International conference on medical image computing and computer-assisted intervention. Springer; 2015. p. 677–684.

[18] Kawata Y, Arimura H, Yamashita Y, Magome T, Ohki M, Toyofuku F, Higashida Y, Tsuchiya K (2010). Computer-aided evaluation method of white matter hyperintensities related to subcortical vascular dementia based on magnetic resonance imaging. Comput Med Imaging Graph.

[19] Qin C, Moreno RG, Bowles C, Ledig C, Scheltens P, Barkhof F, Rhodius-Meester H, Tijms B, Lemstra AW, Van Der Flier, WM, et al. A semi-supervised large margin algorithm for white matter hyperintensity segmentation. In: International workshop on machine learning in medical imaging. Springer; 2016. p. 104–112.

[20] Ghafoorian M, Karssemeijer N, Heskes T, Van Uder I, de Leeuw F-E, Marchiori E, van Ginneken B, Platel B. Non-uniform patch sampling with deep convolutional neural networks for white matter hyperintensity segmentation. In: 2016 IEEE 13th international symposium on biomedical imaging (ISBI). IEEE; 2016. p. 1414–1417.

[21] Han Z, Wei B, Hong Y, Li T, Cong J, Zhu X, Wei H, Zhang W (2020). Accurate screening of covid-19 using attention based deep 3d multiple instance learning. IEEE Trans Med Imaging.

[22] Li T, Wei B, Cong J, Li X, Li S (2019). S 3 egANet: 3D spinal structures segmentation via adversarial nets. IEEE Access.

[23] Guerrero R, Qin C, Oktay O, Bowles C, Chen L, Joules R, Wolz R, Valdés-Hernández MDC, Dickie D, Wardlaw J (2018). White matter hyperintensity and stroke lesion segmentation and differentiation using convolutional neural networks. NeuroImage Clin.

[24] Brosch T, Yoo Y, Tang LY, Li DK, Traboulsee A, Tam R. Deep convolutional encoder networks for multiple sclerosis lesion segmentation. In: International conference on medical image computing and computer-assisted intervention. Springer; 2015. p. 3–11.

[25] Kamnitsas K, Ledig C, Newcombe VF, Simpson JP, Kane AD, Menon DK, Rueckert D, Glocker B (2017). Efficient multi-scale 3D CNN with fully connected CRF for accurate brain lesion segmentation. Med Image Anal.

[26] Ghafoorian M, Karssemeijer N, Heskes T, van Uden IW, Sanchez CI, Litjens G, de Leeuw F-E, van Ginneken B, Marchiori E, Platel B (2017). Location sensitive deep convolutional neural networks for segmentation of white matter hyperintensities. Sci Rep.

[27] Chen X, Kundu K, Zhu Y, Berneshawi AG, Ma H, Fidler S, Urtasun R. 3d object proposals for accurate object class detection. In: Advances in neural information processing systems; 2015. p. 424–432.

[28] Cao G, Xie X, Yang W, Liao Q, Shi G, Wu J. Feature-fused SSD: fast detection for small objects. In: Ninth international conference on graphic and image processing (ICGIP 2017). International Society for Optics and Photonics; 2018. p. 10615.

[29] Yang F, Choi W, Lin Y. Exploit all the layers: Fast and accurate CNN object detector with scale dependent pooling and cascaded rejection classifiers. In: Proceedings of the IEEE conference on computer vision and pattern recognition; 2016. p. 2129–2137.

[30] Li J, Liang X, Wei Y, Xu T, Feng J, Yan S. Perceptual generative adversarial networks for small object detection. In: Proceedings of the IEEE conference on computer vision and pattern recognition; 2017. p. 1222–1230.

[31] Eggert C, Zecha D, Brehm S, Lienhart R. Improving small object proposals for company logo detection. In: Proceedings of the 2017 ACM on international conference on multimedia retrieval; 2017. p. 167–174.

[32] Ren Y, Zhu C, Xiao S (2018). Small object detection in optical remote sensing images via modified faster R-CNN. Appl Sci.

[33] Ronneberger O, Fischer P, Brox T. U-net: convolutional networks for biomedical image segmentation. In: International conference on medical image computing and computer-assisted intervention. Springer; 2015. p. 234–241.

[34] Tieleman T, Hinton G (2012). Lecture 6.5-rmsprop: divide the gradient by a running average of its recent magnitude. COURSERA Neural Netw Mach Learn.

[35] Buda M, Maki A, Mazurowski MA (2018). A systematic study of the class imbalance problem in convolutional neural networks. Neural Netw.

[36] Kohavi R, et al. A study of cross-validation and bootstrap for accuracy estimation and model selection. In: Ijcai, Montreal, Canada; 1995;14:1137–1145.

